# Determination of adrenal hypersecretion in primary Aldosteronism without aldosterone-production adenomas

**DOI:** 10.1186/s12902-021-00770-1

**Published:** 2021-05-31

**Authors:** Fang Sun, Yangning Hong, Hexuan Zhang, Xiaoli Liu, Zhigang Zhao, Hongbo He, Zhencheng Yan, Zhiming Zhu

**Affiliations:** Center for Hypertension and Metabolic Diseases, Department of Hypertension and Endocrinology, Daping Hospital, Army Medical University of PLA, Chongqing, 400042 China

**Keywords:** Adrenal vein sampling, Primary aldosteronism, Unilateral aldosterone hypersecretion

## Abstract

**Background:**

Primary aldosteronism (PA) is highly prevalent in hypertensive population. Adrenal vein sampling (AVS) is the only procedure to assess adrenal aldosterone hypersecretion in PA. PA patients without aldosterone-producing adenomas (APA) frequently have unilateral aldosterone hypersecretion (UAH). These patients could bear inappropriate adrenalectomy without AVS. This study aims to identify which clinical characteristics should be recommended to perform AVS in these PA patients.

**Methods:**

This study was performed from January 2018 to July 2019 at a center for hypertension and metabolic diseases. Adrenal computed tomography (CT) scan, biochemical evaluation, and AVS were performed.

**Results:**

Total 141 patients were included in this study. Aldosterone to renin ratio (ARR) after confirmatory test is highly associated with adrenal laterality. The specificity of ARR > 10 (ng/dL)/(mU/L) after confirmatory test is 100%. After confirmatory test, patients with ARR > 10 (ng/dL)/(mU/L) had higher plasma aldosterone concentration and incidences of ischemic heart diseases and renal damage(*p* < 0.05).

**Conclusions:**

After confirmatory tests, ARR > 10 (ng/dL)/(mU/L) indicates adrenal laterality, with increasingly cardiorenal damage in PA patients without APA. Thus, AVS should be recommended in these patients before surgery.

**Trial registration:**

NCT03398785, Date of Registration: December 24, 2017.

**Supplementary Information:**

The online version contains supplementary material available at 10.1186/s12902-021-00770-1.

## Background

Primary aldosteronism (PA) is characterized by excessive aldosterone produced by adrenals. PA is the most common cause of endocrine hypertension and is prevalent in approximately 10–15% in resistant hypertension [1, 2]. Excessive aldosterone remarkably increases the risks of cardiovascular diseases and stroke [3–5]. Patients with unilateral adrenal hypersecretion (UAH) could be cured by unilateral adrenalectomy [6, 7]. Moreover, recent studies showed that long-term administration of MR antagonists did not remit the cardiovascular damage in PA patients compared to that in blood pressure-matched hypertensive patients [8].

Adrenal computed tomography (CT) imaging can show adrenal adenoma, but cannot distinguish nonfunctioning incidentalomas [9, 10]. Currently, adrenal venous sampling (AVS) is the only procedure to identify UAH; however, it is available only in few referral centers worldwide because its technique is challenging [11–13]. The discordance between CT imaging and AVS is higher in PA patients with normal adrenal imaging and UAH or PA patients with unilateral nodules and isolated contralateral aldosterone hypersecretion [9, 14–16]. Obviously, it is impossible to identify these adrenal hypersecretions without AVS.

Endocrine society clinical practice guidelines recommend that patients with established PA should undergo AVS when adrenalectomy is considered [17]. AVS can be spared if patients are aged < 35–40 years with marked PA and have a clear unilateral adrenal adenoma and a normal contralateral adrenal gland [18–21]. However, these clinical parameters are not applicable for patients without APA. Therefore, selective AVS is essential for appropriate examination in UAH patients without APA. At present, it is unclear what are the clinical characteristics in UAH patients without APA. The purpose of this study was to determine whether lateralized AVS can be predicted from clinical, biochemical, and imaging characteristics in these patients.

## Methods

### Study population

Of the total 2753 hypertensive patients referred to our center for screening PA from January 2018 to July 2019, we retrospectively reviewed the AVS data of 292 patients who were suspected to have secondary hypertension and excluded 14 patients who failed in AVS, 17 patients with Cushing syndrome / Adrenal medullary hyperplasia, 80 patients with Conn syndrome, and 40 patients without complete biochemical result. The remaining 141 patients were analyzed for the following parameters: age, sex, body mass index (BMI), serum potassium, serum sodium, urinary potassium, urinary sodium, estimated glomerular filtration rate (eGFR), renin, aldosterone, aldosterone to renin ratio (ARR) value after the confirmation test, CT imaging, and AVS examination. Blood pressure was obtained in three consecutive measurements after taking a 5-min rest. Blood samples were obtained in the sitting/recumbent position during 8:00 am to 10:00 am. All antihypertensive drugs that can interfere with the renin-angiotensin-aldosterone system were discontinued for at least 2–4 weeks before the screening test. Patients with hypokalemia were treated with potassium sparing agents. Inclusion criteria— mainly referred to Endocrine Society guideline [17]: ① Blood pressure ≥ 140/90 mmHg or under treatment, and/or hypokalemia (serum potassium ≤3.5 mmol/L); ②Elevated ARR; ③Suspicious positive or positive results of confirmatory test, confirmatory test is excluded if clinical symptoms are typical, renin is inhibited and PAC > 20 ng/dL; ④ CT indicates normal appearing adrenals, minimal unilateral adrenal limb thickening, unilateral microadenomas (≤1 cm), or bilateral macro- or microadenomas (or a combination of the two). Exclusion criteria: ①APA: unilateral macroadenoma (diameter > 1 cm) and the opposite side was normal [18, 22, 23];②Aldosterone-producing adrenal carcinomas (diameter > 4 cm), glucocorticoid remediable aldosteronism (GRA) and familial hyperaldosteronism;③Any other secondary hypertension: renal parenchymal hypertension, renal artery stenosis, Cushing’s syndrome, adrenal medullary hyperplasia, coarctation of aorta, obstructive sleep apnea hypopnea syndrome, etc.;④AVS procedure failed. Major complications: ①Ischemic heart disease [22]: (1) typical clinical symptoms; (2) new ischemic ECG changes; (3) new pathological Q waves; (4) new imaging evidence of myocardial loss or abnormal ventricular wall motion; (5) coronary angiography or intracavitary imaging abnormalities or autopsy confirmed coronary thrombus; ②Hypertensive Nephropathy [24]: (1) hypertension; (2) microalbuminuria> 20 mg/d or ACR > 30 mg/mmol; (3) excluded due to renal parenchymal diseases (glomerulopathy or tubulopathy diseases). This study complied with the tenets of the Declaration of Helsinki and was approved by the ethics committee of institute. Written informed consent was obtained from all patients.

### Adrenal vein sampling

AVS has been performed through the upper extremity route (median cubital vein or basilic vein). Briefly, A French 6 sheath was inserted through the percutaneous forearm vein approach. A 5F MPA (125 cm, Cook Medical, Bloomington, IN, USA) catheters were advanced slowly to the right atrium, then into the inferior vena cava (IVC), bypassing the right ventricle. The right adrenal vein was catheterized, and the position of the catheter tip was verified by gentle injection of a small amount of nonionic contrast medium and documented radiographically. Blood was obtained from the right adrenal vein. Then, a TIG catheter (Terumo Corporation, Tokyo, Japan) was used to catheterize the left adrenal vein. Heparin (50 U/Kg) was administered to avoid adrenal venous thrombosis during AVS procedure [25, 26].

Successful sampling was determined by high selectivity index (cortisol in the adrenal vein/ cortisol ≥2 subclavian vein without adrenocorticotropic hormone simulation). To correct for the dilution effect of the inferiorphrenic vein flow into the common phrenic trunk, the right and left adrenal vein aldosterone concentrations were divided by the respective cortisol concentrations. Plasma cortisol was used to calibrate the aldosterone level to obtain the local standardized concentration of aldosterone. The catheterization was successful if the concentration of intravenous cortisol / inferior vena cava cortisol (SI) ≥ 2 and a lateralization index≥2 was indicated the unilateral aldosterone hypersecretion based on an expert consensus on the use of AVS for PA subtype [17].

### Confirmatory test

Captopril challenge test (CCT) and saline infusion test (SIT) were used as confirmatory tests for PA diagnosis. Aldosterone and renin level were tested by Chemiluminescence Immunoassay (CLIA) (DiaSorin, Saluggia, Italy). Positive results were determined according to Endocrine Society guideline [17].

### Statistical analysis

IBM SPSS Statistics 20 was used for statistical analyses. We used F-test or Kruskal–Wallis test to compare various parameters according to the data distribution. Quantitative values between groups were compared by *t* test and the Mann-Whitney test, while Chi-square test was used for categorical variables. Variables associated with lateralized AVS in the bivariate analysis (*p* < 0.05) were entered in logistic regression models, and stepwise forward selection was performed to find whether variables were independently associated with lateralized AVS (Wald test *p* < 0.1). C-Statistic was used to evaluate the discrimination ability of regression models. *p* < 0.05 was considered statistically significant.

## Results

### Screening process of patients

**Fig. 1:**
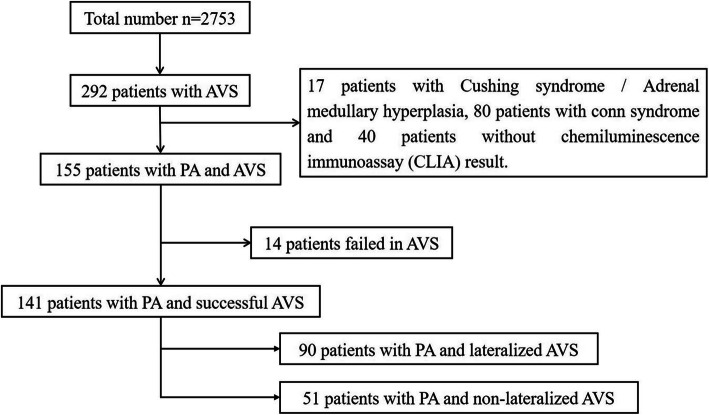
Patient selection and distribution. Adrenal vein catheterization was considered successful when selectivity index (SI) was ≥2 without cosyntropin administration. Unilateral PA was diagnosed if lateralized index (LI) was ≥2. Abbreviation:PA, primary aldosteronism; AVS, Adrenal vein sampling

Database retrieval yielded 292 AVS in 2753 patients during the study period (Fig. 1). Of these 292 patients, 97 (33.2%) did not fulfill the inclusion criteria, 40 (13.7%) were without complete biochemical result, and 14 (4.8%) had a failure of AVS. The remaining 141 patients were included: 90 (63.8%) had a lateralization; 51 (36.2%) had non-lateralized AVS. Among them, 33 patients met the criteria for spare confirmatory tests. Among the remaining 108 patients, 25(23.1%) underwent SIT and 83(76.9%) underwent CCT.

### Baseline characteristics of patients with AVS subtype

According to the presence or absence of lateralization examined by AVS, patients were divided into lateralized PA and non-lateralized PA. Except for the difference in the lateralization ratio, there was no difference in other clinical parameters and proportion of complications between the two groups (Table 1).

**Table 1:** Baseline characteristics of included patients

Variable	Lateralized ***N*** = 90	Non-lateralized ***N*** = 51	***P*** value
Lateralization index	12.1 ± 28.0	1.5 ± 0.4	< 0.001
Mean Age	49.2 ± 10.3	50.0 ± 10.0	0.88
Sex, M/F(n%)	44 (48.9%)/46 (52.2%)	22 (43.1%)/29 (56.9%)	0.51
BMI (Kg/m^2^)	25.9 ± 2.7	25.2 ± 3.7	0.07
Systolic BP (mmHg)	145 ± 19	144 ± 21	0.32
Diastolic BP (mmHg)	91 ± 13	90 ± 15	0.24
Serum potassium (mmol/L)	3.6 ± 0.5	3.7 ± 0.5	0.98
Serum sodium (mmol/L)	137.1 ± 21.1	140.1 ± 2.1	0.07
Urinary potassium (mmol/d)	40.1 ± 15.9	37.8 ± 14.5	0.64
Urinary sodium (mmol/d)	181.5 ± 108.8	168.8 ± 97.8	0.75
Recumbent PAC (ng/dL)	11.9 [8.9–18.1]	11.1 [8.4–16.5]	0.70
Recumbent DRC (mU/L)	1.6 [0.9–5.0]	2.9 [1.3–5.6]	0.07
Recumbent ARR (ng/dL)/(mU/L)	4.8 [1.9–8.9]	3.1 [1.7–5.9]	0.19
Post-CCT/SIT ARR (ng/dL)/(mU/L)	2.9 [1.05–6.90]	2.2 [1.0–4.0]	0.24
ACR (mg/mmol)	118.7.1 ± 428.2	44.2 ± 76.5	0.09
Serum creatine (umol/L)	68.7 ± 23.5	70.4 ± 48.5	0.24
eGFR (ml/min/1.73 m^2^)	131.7 ± 41.0	137.0 ± 33.2	0.21
Hypertensive heart disease, n(%)	6 (8.8%)	2 (5.1%)	0.32
Ischemic heart disease, n(%)	31 (44.9%)	13 (33.3%)	0.71
Hypertensive Nephropathy,n(%)	26 (42.6%)	10 (27.8%)	0.31
Peripheral vascular disease, n(%)	11 (16.7%)	2 (5.1%)	0.19
Cerebrovascular disease, n(%)	27 (50.0%)	18 (51.4%)	0.13
Metabolic syndrome, n(%)	6 (8.8%)	2 (5.1%)	1.0

Continuous variables are expressed as the mean ± SD or the medians [interquartile range] unless noted otherwise

*Abbreviations*: *LI* Lateralization index, *BMI* body mass index, *CCT* captopril challenge test, *SIT* saline infusion test, *ARR* ratio of aldosterone/renin, *PAC* plasma aldosterone concentration, *DRC* direct renin concentration, *ACR* urinary albumin/creatinine ratio

### Logistic model predicting adrenal lateralization

Age, serum potassium, eGFR, PAC, and ARR after confirmatory tests were used to distinguish the lateralized AVS in patients with UAH. Thus, a multivariable logistic regression model was used to predict the lateralized AVS based on these variables (Table 2). This model has statistical significance (χ^2^ = 7.519 *p* < 0.01) and good fitting effect(χ^2^ = 10.79 *p* = 0.21). Only one combination variable was entered in the model: ARR after confirmatory tests (the coefficients: 1.010, 95% CI: 1.000 to 1.021, *p* < 0.044). 13.9% (15 of 108) of PA patients without APA showed a sensitivity of 22.1%, a specificity of 100%, while the specificity of serum potassium and PAC was lower (Table 3). In addition, several models for predicting AVS avoidance reported by other studies were used to evaluate our results. It showed a poor prediction (Supplementary Table S1).

**Table 2:** Multivariable logistic regression analyzed for five variable

Variable	B	SE	Wald	***P***value	OR	95%CI
ARR after CCT/SIT	0.01	0.005	4.071	0.044	1.010	1.000 to 1.021
Age	N/A	N/A	N/A	0.829	N/A	N/A
Serum potassium	N/A	N/A	N/A	0.722	N/A	N/A /
eGFR	N/A	N/A	N/A	0.898	N/A	N/A
PAC	N/A	N/A	N/A	0.528	N/A	N/A

*P* value < 0.05 is considered statistically significant. Stepwise method with all relative parameters as independent variables including age, serum potassium, eGFR, PAC, and ARR after confirmatory test. Boldface indicates the factor with significant association (*p* < 0.05)

*Abbreviations*: *B* regression coefficient, *SE* standard error, *OR* Odds ratio, *CI* confidence interval, *CCT* captopril challenge test, *SIT* saline infusion test, *PAC* Plasma aldosterone concentration, *ARR* aldosterone to renin ratio, *eGFR* estimated glomerular filtration rate, *N/A* not applicable

**Table 3:** Sensitivity and specificity of various parameters

Diagnostic criteria	N	Sensitivity	Specificity
PAC > 171 ng/dL	32/141	26.7%(17.8–37.4)	84.3%(71.2–92.2)
Serum potassium< 3.5 mmol/L	54/141	36.7%(26.8–47.5)	58.8%(44.2–72.4)
Post-CCT/SIT ARR > 10(ng/dL)/(mU/L)	15/108	22.1%(12.9–33.8)	100%(93.2–100.0)
Hypertension; Serum potassium< 3.5 mmol/L;Post-CCT/SIT ARR > 10(ng/dL)/(mU/L)	9/108	13.0%(6.1–23.3)	100%(91.0–100.0)
Serum potassium< 3.5 mmol/L or Post-CCT/SIT ARR > 10(ng/dL)/(mU/L)	51/108	49.3%(37.0–61.6)	56.4%(39.6–72.2)

The data are expressed as n(%) unless noted otherwise;

*Abbreviations*: *CCT* captopril challenge test, *SIT* saline infusion test, *ARR* ratio of aldosterone/renin, *PAC* plasma aldosterone concentration

### Association between ARR and target organ damage

We further examined whether UAH patients with ARR > 10 (ng/dL)/(mU/L) were susceptible to target organ damage and PA-associated complications (Supplementary Table S2). The PAC was significantly higher, while direct renin concentration was significantly lower in UAH patients with ARR > 10 (ng/dL)/(mU/L) compared to that in UAH patients with ARR ≤10 (ng/dL)/(mU/L) (*p* < 0.01). However, serum potassium level was not different between the two groups. Importantly, the incidence of ischemic heart diseases and hypertensive nephropathy was higher in UAH patients with ARR > 10 (ng/dL)/(mU/L) (*p* < 0.05). Correlation analysis showed a significant association between PAC and aldosterone concentration of the adrenal vein (*r* = 0.299 *p* < 0.01), and serum creatinine level and ACR (urinary albumin/creatinine ratio) were significantly correlated with the PAC (*r* = 0.218 *p* < 0.05, *r* = 0.233 *p* < 0.05), respectively (Supplementary Table S3).

## Discussion

The major findings of this study are that: 63.8% patients without APA have UAH. ARR after confirmatory test can predict lateralized well, with very high specificity. After confirmatory test, UAH patients with higher ARR are susceptible to target organ damage. Thus, AVS was strongly recommended before surgery in these patients.

A recent study by Brown and colleagues reported that the prevalence of unrecognized PA was about 16–22% in general hypertension population [27]. Identifying adrenal hypersecretion is critical to hypertension control and remit target organ damage in PA. Currently, secondary causes associated with resistant hypertension, such as PA, has been included in the American Heart Association guideline [28]. Therefore, laparoscopic adrenalectomy is recommended for PA patients with APA or UAH. Nevertheless, adrenal CT image is unreliable to identify adrenal hypersecretion. Young et al. reported that most patients with UAH have normal adrenal imaging or unilateral micronodule but contralateral adrenal hypersecretion [22]. Moreover, a systematic review indicated that CT/MRI results did not agree with AVS results in 37.8% of patients. If only CT imaging is used to determine adrenal lateralization, 14.6 and 19.1% patients would bear inappropriate adrenalectomy or inappropriate exclusion from surgery, and surgery on the wrong adrenal side would have occurred in 3.9% [29]. Although some studies showed that the use of ACTH during AVS was helpful for improving the success rate [30, 31]. However, a recent study suggested that stimulated AVS with ACTH did not contribute to better clinical and biochemical outcomes [32]. In this study, we showed 63.80% PA patients without APA had UAH through AVS without ACTH stimulation. We indicate that a considerable proportion of PA patients without APA is worthy to consider AVS examination. However, it is impractical to perform AVS universally in PA patients because this procedure is an invasive and technically demanding [22, 33]. Several clinical models are applied to predict which PA patients should spare AVS [18, 34, 35], but the prediction results were not optimal when we used these models in our patients. More importantly, these prediction models cannot distinguish UAH. In clinical practice, it is difficult to judge which lateralized characteristic emerges in UAH patients without APA.

The present study showed that ARR after confirmatory test had significant difference between PA patients with and without AVS laterality. Furthermore, the specificity of ARR > 10 (ng/dL)/(mU/L) after confirmatory tests can reach 100% in PA patients with laterality. Therefore, ARR after confirmatory test can distinguish which PA patient has a strong AVS indication.

It is well known that the excessive aldosterone causes cardiovascular and renal damage from both experimental and human studies. It shows that the target organ damage is more remarkable in patients with PA compared to those with essential hypertension [3–5]. In this study, we showed that PA patients with ARR > 10 (ng/dL)/(mU/L) after confirmatory tests had higher plasma aldosterone concentration and more severe cardiorenal complications than the control group. Furthermore, there was an association between plasma aldosterone concentration and renal dysfunction. Hence, it is essential to eliminate the excessive aldosterone in these patients through surgery.

The present study showed that the higher specificity of our prediction value suggests that only small portions of UAH patients without APA are recommended to undergo AVS. These results also are consistent with the consensus recommended by Endocrinology Society guideline. The strengths of this study is that we provide a simple method to evaluate whether adrenal lateralization exists in PA patients without apparent adenomas, and further suggest patients to consider the adrenalectomy. The weakness of this study is a relatively small sample in a single center. Therefore, the positive predictive value of ARR needs to be verified by other investigator.

## Conclusions

Considerable PA patients without APA have unilateral adrenal hypersecretion. After confirmatory tests, ARR > 10 (ng/dL)/(mU/L) indicates the adrenal laterality, which increases cardiorenal damage in these patients. Thus, AVS should be recommended in these patients before surgery.

## Supplementary Information

**Additional file 1: Table S1.**Comparison of different clinical prediction scores in PA patients with and without ARR greater than 10 after confirmatory test.

**Additional file 2: Table S2.**Comparison of target organ damage in PA patients with and without ARR greater than 10 after confirmatory test (ng/dL)/(mU/L).

**Additional file 3: Table S3.** The correlation between PAC and the laboratory indexes of target organ damage.

## Data Availability

All data generated or analyzed during this study are included in this published article and information about experimental sessions and results are available from the corresponding author on reasonable request.

## Abbreviations

CCT: Captopril challenge test
SIT: Saline infusion test
ARR: Ratio of aldosterone/renin
PAC: Plasma aldosterone concentration
PA: Primary aldosteronism
AVS: Adrenal vein sampling
LI: Lateralization index
BMI: Body mass index
DRC: Direct renin concentration
ACR: Urinary albumin/creatinine ratio
