# Supplementary material for: Determination of adrenal hypersecretion in primary Aldosteronism without aldosterone-production adenomas
Source: BMC Endocr Disord. 2021 May 31;21:114. doi: 10.1186/s12902-021-00770-1 (PMC8167985; doi:10.1186/s12902-021-00770-1)
Supplement: Supplementary file 1 — Additional file 1: Table S1.Comparison of different clinical prediction scores in PA patients with and without ARR greater than 10 after confirmatory test. [file 12902_2021_770_MOESM1_ESM.docx]

| **Table S1.**Comparison of different clinical prediction scores in PA patients with and without ARR greater than 10 after confirmatory test | | | |
| --- | --- | --- | --- |
|  | **Post-CCT/SITARR>10**  **(ng/dL)/(mU/L) (n=15)** | **Post-CCT/SITARR≤10**  **(ng/dL)/(mU/L)**  **(n=40)** | **Reference range** |
| Kuper score* | 3.00$\pm$1.13 | 2.35$\pm$0.92 | 0-7 |
| Modified Kupers score* | 3.20$\pm$1.01 | 2.53$\pm$0.88 | 0-7 |
| Nanba score* | 2.93$\pm$1.98 | 1.18$\pm$1.66 | 0-8 |

References cited(18,30,31)

Abbreviations: CLIA, Chemiluminescence Immunoassay; SIT, saline infusion test; ARR aldosterone to renin ratio.
