# Supplementary material for: Determination of adrenal hypersecretion in primary Aldosteronism without aldosterone-production adenomas
Source: BMC Endocr Disord. 2021 May 31;21:114. doi: 10.1186/s12902-021-00770-1 (PMC8167985; doi:10.1186/s12902-021-00770-1)
Supplement: Supplementary file 2 — Additional file 2: Table S2.Comparison of target organ damage in PA patients with and without ARR greater than 10 after confirmatory test (ng/dL)/(mU/L). [file 12902_2021_770_MOESM2_ESM.docx]

| **Table S2.**Comparison of target organ damage in PA patients with and without ARR greater than 10 after confirmatory test(ng/dL)/(mU/L) | | | |
| --- | --- | --- | --- |
| **Variable** | **post-CCT/SITARR>10**  **(ng/dL)/(mU/L)**  **n=15** | **post-CCT/SITARR≤10**  **(ng/dL)/(mU/L)**  **n=93** | ***p* value** |
| Sex,M/F,n% | 8(53.3%)/7(46.7%) | 40(43.0%)/53(57.0%) | 0.455 |
| Age(y) | 53.0±11.3 | 50.1±9.8 | 0.702 |
| Duration(y) | 7(5-20) | 3(1-10) | 0.084 |
| Body mass index(Kg/m^2^) | 25.7$\pm$2.3 | 25.7$\pm$3.3 | 0.178 |
| Systolic BP(mmHg) | 149$\pm$17 | 147$\pm$20 | 0.375 |
| Diastolic BP(mmHg) | 90$\pm$14 | 91$\pm$13 | 0.761 |
| Recumbent PAC (ng/dL) | 21.4[12.7-35.7] | 11.10[8.0-16.0] | **0.001** |
| Recumbent DRC (mU/L) | 0.84[0.66-1.06] | 2.39[1.41-5.07) | **0.003** |
| Recumbent ARR(ng/dL)/(mU/L) | 2.5[1.5-5.0] | 4.2[2.2-7.1] | **<0.001** |
| Serum potassium, (mmol/L) | 3.4$\pm$0.4 | 3.6$\pm$0.5 | 0.671 |
| Urinary potassium, (mmol/d) | 43.6$\pm$11.0 | 41.1$\pm$16.8 | 0.166 |
| IGT or DM, n (%) | 3(20.0%) | 31(33.3%) | 0.302 |
| Hypertensive heart disease, (%) | 3(23.1%) | 20(23.3%) | 0.989 |
| Ischemic heart disease, (%) | 4(36.4%) | 4(4.3%) | **0.001** |
| Hypertensive Nephropathy, (%) | 10(66.7%) | 34(36.6%) | **0.028** |
| Peripheral vascular disease, (%) | 5(41.7%) | 31(36.5%) | 0.727 |
| Cerebrovascular disease, n(%) | 3(25.0%) | 10(20.8%) | 0.158 |
| MS, n(%) | 6(54.5%) | 39(50.0%) | 0.778 |
| UMA (mg/24h) | 48.2$\pm$77.9 | 41.5$\pm$152.6 | 0.811 |
| ACR(mg/mmol) | 121.1$\pm$140.1 | 86.5$\pm$348.9 | 0.982 |
| Serum creatine (umol/L) | 72.8$\pm$28.4 | 64.3$\pm$20.1 | 0.114 |
| eGFR (ml/min/1.73 m^2^) | 122.7$\pm$49.6 | 139.2$\pm$36.4 | 0.147 |

Continuous variables are expressed as the mean$\pm$SD or the median [interquartile range] unless noted otherwise. ^a^Comparison across groups was performed by Mann-Whitney U test or t-test (*P* represent across group test).

Abbreviations: PAC, Plasma aldosterone concentration; DRC, Direct renin concentration; ARR, aldosterone to renin ratio; IGT, impaired glucose tolerance; DM, diabetes mellitus; MS, metabolic syndrome; UMA, urine microalbuminuria; ACR, urinary albumin/creatinine ratio.

By analyzing the correlation between the local or peripheral aldosterone PAC level and the target organ damage indexes in 108 patients who whether meet the elevated ADRR after confirmatory test or not, we can conclude that although there is a weak correlation between the aldosterone concentration of peripheral and local adrenal vein (r=0.299 p<0.01), the renal damage indexes including serum creatinine and eGFR are significantly correlated with the peripheral PAC (*r*= 0.218 *p*<0.05, *r*=0.233 *p*<0.05, respectively).
