# Supplementary material for: Determination of adrenal hypersecretion in primary Aldosteronism without aldosterone-production adenomas
Source: BMC Endocr Disord. 2021 May 31;21:114. doi: 10.1186/s12902-021-00770-1 (PMC8167985; doi:10.1186/s12902-021-00770-1)
Supplement: Supplementary file 3 — Additional file 3: Table S3. The correlation between PAC and the laboratory indexes of target organ damage. [file 12902_2021_770_MOESM3_ESM.docx]

| **Table S3.** The correlation between PAC and the laboratory indexes of target organ damage | | | | | | | | | | | |
| --- | --- | --- | --- | --- | --- | --- | --- | --- | --- | --- | --- |
| **Item** | **LAD**  **(mm)** | **LVDd**  **(mm)** | **IVST**  **(mm)** | **PWT**  **(mm)** | **E/A**  **(mm)** | **EF%**  **(mm)** | **SCr(umol/liter)** | **eGFR (ml/min^-1^·1.73 m2^-1^)** | **UMA**  **(mg/24h)** | **ACR**  **(mg/mmol)** | **Local PAC**  **(ng/dl)** |
| Peripheral PAC(ng/dl) | *r*=-0.107  *p*=0.335 | *r*=0.090  *p* =0.420 | *r*=0.046  *p* =0.679 | *r*=-0.054  *p* =0.62 | *r* =-0.214  *p* =0.055 | *r* =0.030  *p* =0.788 | ***r* =0.218**  ***p* =0.023** | *r* =-0.149  *p=* 0.123 | *r* =0.184  *p* =0.061 | ***r* =0.233**  ***p* =0.017** | ***r* =0.299**  ***p* =0.002** |
| Local PAC(ng/dl) | *r* =0.085  *p* =0.443 | *r* =0.121  *p* =0.278 | *r* =0.034  *p* =0.759 | *r* =0.009  *p* =0.938 | *r* =-0.131  *p* =0.244 | *r* =0.091  *p* =0.451 | *r* =0.186  *p* =0.054 | *r* =-0.121  *p* =0.213 | *r* =0.118  *p* =0.241 | *r* =0.141  *p* =0.153 | / |

Abbreviations: CLIA, Chemiluminescence Immunoassay; LAD, Left Atria Diameter; LVDd, Left Ventricular end Diastolic dimension; IVST, Interventricular Septum Thickness; PWT, Posterior Wall Thickness; E/A, mitral inflow E/A ratio; EF, Ejection Fraction. SCr, Serum Creatine; eGFR, Estimated glomerular filtration rate; UMA, urine microalbuminuria; ACR, urinary albumin/creatinine ratio;
